# Insights into the pathogenesis of axial spondyloarthropathy from network and pathway analysis

**DOI:** 10.1186/1752-0509-6-S1-S4

**Published:** 2012-07-16

**Authors:** Jing Zhao, Jie Chen, Ting-Hong Yang, Petter Holme

**Affiliations:** 1 Department of Mathematics, Logistical Engineering University, Chongqing 400016, China; 2 Department of Natural Medicinal Chemistry, Second Military Medical University, Shanghai, China; 3 Department of Mathematics and Statistics, University of Missouri-Kansas City, MO 64110-2499, USA; 4 Department of Physics, Umeå University, 90187 Umeå, Sweden; 5 Department of Energy Science, Sungkyunkwan University, Suwon 440-746, Korea

## Abstract

**Background:**

Complex chronic diseases are usually not caused by changes in a single causal gene but by an unbalanced regulating network resulting from the dysfunctions of multiple genes or their products. Therefore, network based systems approach can be helpful for the identification of candidate genes related to complex diseases and their relationships. Axial spondyloarthropathy (SpA) is a group of chronic inflammatory joint diseases that mainly affect the spine and the sacroiliac joints. The pathogenesis of SpA remains largely unknown.

**Results:**

In this paper, we conducted a network study of the pathogenesis of SpA. We integrated data related to SpA, from the OMIM database, proteomics and microarray experiments of SpA, to prioritize SpA candidate disease genes in the context of human protein interactome. Based on the top ranked SpA related genes, we constructed a SpA specific PPI network, identified potential pathways associated with SpA, and finally sketched an overview of biological processes involved in the development of SpA.

**Conclusions:**

The protein-protein interaction (PPI) network and pathways reflect the link between the two pathological processes of SpA, i.e., immune mediated inflammation, as well as imbalanced bone modelling caused new boneformation and bone loss. We found that some known disease causative genes, such as TNFand ILs, play pivotal roles in this interaction.

## Background

Axial spondyloarthropathy (SpA) is a family of chronic inflammatory joint diseases of the spine and the sacroiliac joints. One of the major prototypes of SpA is ankylosing spondylitis (AS). The two central features of SpA are inflammation and new bone formation, especially in the spine [1]. The inflammation first occurs around the sites where ligaments attach to the bone. As the inflammation heals, there is new bone formation in the ligament, causing the thickening or hardening of the underlying bone, and eventually the fusion of the vertebral bodies and even the spinal stiffness. It is known that SpA is associated with multiple genes, such as HLA-B27, TNF and IL23R [2]. However, the pathogenesis of SpA remains largely unknown. The complexity of the disorder indicates a multifactorial etiology involving multiple biological processes or pathways.

The pathogenesis of complex chronic diseases such as SpA is believed to happen due to the malfunction of multiple genes and gene products. It has been fairly well confirmed that the propensity of many diseases can be reflected by altered gene and protein expression levels in particular cell types [3,4]. High throughput experiments at transcriptomic and proteomic levels have been applied to screen potentially disease associated factors of SpA. Some genes involved in the innate immune system, such as SPARC, SLPI and NLRP2, and proteins known to be tumor necrosis factor (TNFa)inducible were identified to be up-expressed in SpA [4-7]. On the other hand, disease associated genes tend to share common functional features, be co-expressed in specific tissues, and their protein products have a tendency to interact with each other [8]. Several computational methods have been developed accordingly to predict disease associated genes based on PPI [9-12], or the integration of gene expression data with PPI [13-15]. Furthermore, research has been conducted trying to identify pathogenic processes by the integrated computational analysis of heterogeneous data sources, including genetics, transcriptomics, proteomics and interactome data. Many specific disease-associated networks have been constructed, including those related to diabetes mellitus, cancers, asthma, Alzheimer's disease, and cardiovascular diseases [16-27]. In addition, some cellular network or signaling pathway databases have systematically collected pathways associated with specific diseases reported in the literature [28,29]. The disease-associated networks have the promise of allowing for the better understanding of disease pathogenesis, as well as for the identification of potential target sets for therapeutic intervention in the corresponding diseases.

In this work, we integrated SpA-active genes from different resources (known disease genes in the OMIM database [30], proteomic and microarray experiments) and proposed an approach to prioritize candidate genes in the context of human interactome. We then took out the genes most likely associated with SpA to construct a PPI network of SpA and identified potential pathways involved in SpA. Finally, we drew an overview picture of biological processes involved in the development of SpA.

## Results and discussion

### Scoring and ranking genes in the PPI network

Our method to construct disease associated network is based on the observation that proteins coded by genes associated with the same disease tend to be closely located to each other in the protein interaction network [8,31,32]. Starting with a group of SpA-active genes as seeds, we applied a Katz' centrality based index [33] to prioritize candidate genes in the PPI network [15]. Given a weighted human interactome represented as a matrix **W **corresponding to the interaction strength between genes, and a set *D *of *k *known disease-active genes as seeds, we define vector **x **= (*x*_1_, *x*_2_,..., *x_n_*)^T ^as initially known activity of genes in the disease, with *x_i _*= 1 if gene *i *is in the set D, *x_i _*= 0 otherwise. It should be noted that our SpA-active gene set is a combination of proteomics and microarray data, each of which actually has different confidence levels for the study of disease pathogenesis. A good way is to set different weights for them. But determining the value of each weight makes the solution much more difficult. For simplicity, we just give them the same weight in this study.

Let **s **= (*s*_1_, *s*_2_,..., *x_n_*)^T ^be our score vector over the set of genes (where *s_i _*indicates how strong *i *is as a disease-gene candidate),

(1) $$s_{i}^{t+1}=x_{i}+\phi \sum_{j\neq i}w_{ij}s_{j}^{t}$$

where *t *indicates iteration time, and *ϕ *is a parameter that sets the relative contributions of the activity **x **and links in protein interaction networks **W **to the score. If *ϕ *is small, the known activity is more important; if *ϕ *is large, the coupling to the protein neighbours is more important. The parameter *ϕ *needs to be calibrated with real data. The score could be obtained by performing iterations until the algorithm converges, and then all genes in the PPI network could be ranked according to their *s*-scores.

Equation (1) can be represented as matrix form as follows:

(2) s=x+ϕWs

which can be solved by matrix algebra:

(3) $$s={\left[I-\phi W\right]}^{-1}x$$

where [**I**-*ϕ***W**]^-1 ^represents the inverse of the matrix I-*ϕ***W**. We solved equation (3) by Jacobi iteration algorithm.

To get a set of SpA-active genes as seeds, we integrated the results of the proteomics and microarray experiments for SpA and obtained 161 genes potentially active in this disease (See Materials and Methods). Our protein interaction network was constructed from the STRING database [34]. A total of 147 of the 161 genes were found present in the STRING PPI network, and these genes were used as seeds to construct the disease gene activity vector **x **in equation (3).

We searched the Online Mendelian Inheritance in Man (OMIM) database [30] with a keyword "Spondyloarthropathy" and found 7 causal genes with Entrez gene ID. These 7 genes were used to determine the parameter *ϕ*. For each of the genes, we determined a candidate gene set of size *N*(*N *is about 100), including this disease gene, which locate at, or near the cytogentic loci of the SpA-causative gene. For a known SpA-causative gene in a candidate gene set of size *N*, if its *s*-rank calculated by our algorithm is *r*, then *r*-ratio, defined as *r/N*, could reflect how strong this gene is predicted as a disease gene. We determined parameter *ϕ *as the one minimized the average *r*-ratios of the known SpA-causative genes.

For all the genes in the PPI network, we calculated the *s*-score vector by equation (3). Then we ranked the genes in each candidate disease gene set according to their *s*-cores and got their *r*-ratios. We repeated this computation by applying different values of *ϕ *in the area (0, 1/100) and checked the average *r*-ratios of all the 7 known OMIM disease genes. In this way, the best value of *ϕ *was determined as 10^-4^, which minimized the average *r*-ratios of known OMIM disease genes for SpA. For the optimum *ϕ *= 10^-4^, we found an average r-ratio of 0.186, suggesting that the known SpA causative genes were averagely ranked top 18.6% of the candidate genes.

It was known that the performance of our method could be enhanced when partial information about known disease genes was included [15]. Having the optimum value of *ϕ *determined, we added the 7 known SpA-causative genes into the seeds. Then all the genes in the human PPI network were scored according to equation (3).

### PPI network of SpA

The s-score of a gene indicates its possibility associated with the disease. Setting 10% as a cut-off, we took out the genes whose s-scores were top 10% of all genes in the PPI network. We identified a total of 380 genes. Then we limited interactions in the STRING database to weights of at least 0.5, which corresponds to a medium-confidence human genome PPI network [34], and constructed a subnetwork spanned by these 380 genes. Finally, we obtained a PPI network associated with SpA, which included 367 nodes and 8887 edges.

**Figure 1:**
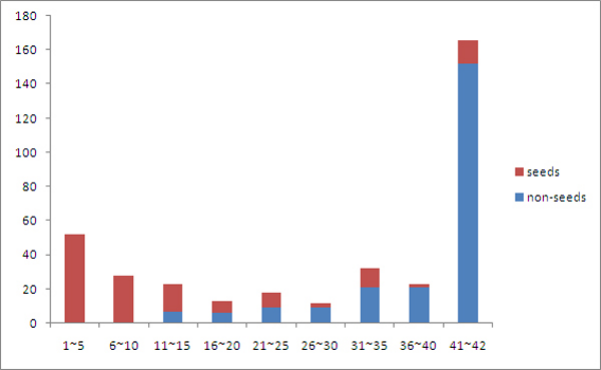
**Distribution of *k*-core layers and seed nodes in the SpA PPI network**.

**Figure 2:**
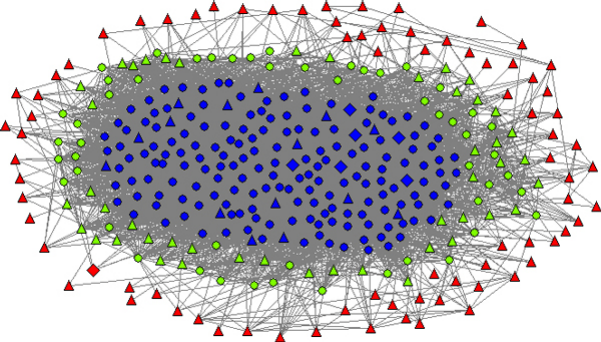
**Core-periphery topology of the SpA PPI network**. Blue, green and red nodes represent the inner core, the medium and outer core layer, respectively. Triangles are seed genes, and diamonds are targets of drugs for SpA.

For 8887 interactions with strength at least 0.5 among only 367 gene-coded proteins, the PPI network of SpA is densely connected. To understand its topology, we conducted a *k*-core decomposition on the network. A so-called *k*-core decomposition is a way to visualize both of the connectivity of neighbourhoods of nodes and their centrality [35]. In short, *k*-core decompositions are obtained by iteratively deleting low-degree nodes to achieve a sequence of *k-cores *(maximal subgraphs with minimal degree *k*, see the Methods section). By and large, following the *k*-core decomposition is similar to zooming into the more central and more interconnected parts of the network. For the PPI network we studied, the innermost core is the 42-core, which includes 143 nodes. In Figure 1, we illustrate the node distribution in the hierarchical *k*-core layers of the PPI network and the number of seed genes in the core layers. This figure suggests that the network topology exhibits a core-periphery dichotomy [36,37] - about half of the nodes in this network interact frequently and are thus interconnected densely to form an inner 36-core, while others communicate with fewer nodes and scatter in different outer core layers to form the periphery of the network. Figure 1 also shows that most seed genes locate at the periphery part whereas the inner core includes most of the non-seed genes. Specifically, a total of 141 seed genes appear in this network, in which only 16 are located in the inner core. All nodes in the core layers 1~10 and about half of the nodes in the core layers 11~35 are seed nodes. Therefore, as shown in Figure 2, we partition the network into three parts accordingly - the inner core is the 36-core, and the medium and outer layers correspond to core layers 11~35 and 1~10, respectively. It can be seen that seed genes in the outer and medium layers tend to interact with genes in the inner core, indicating that the inner core could be the modulating centre of the network.

Furthermore, we searched the DrugBank database [38] for drugs treating SpA and their protein targets. In fact, currently there is no cure for SpA. Three classes of drugs are used clinically for disease management, that is, to reduce inflammation, relieve pain and stiffness, suppress disease activity and slow disease progression [39]. They are non-steroidal anti-inflammatory drug (NSAID), TNF blocker and disease-modifying antirheumatic drug, while the third class shows disappointing effects in practice [39]. We mapped the protein targets of these drugs onto the SpA PPI network and found 7 targets in this network, *i.e*., PTGS2, MMP2, MAPK3, HRAS, EGF, NFKB1 and TNF, in which PTGS2 (Cyclooxygenase 2)and TNF (Tumor necrosis factor) are main therapeutic targets of NSAID and TNF blocker, respectively, TNF and NFKB1 are targets for one of the disease-modifying antirheumatic drugs Thalidomide. TNF is also known disease gene of SpA. PTGS1 (Cyclooxygenase 1), the other therapeutic target of NSAID, does not appear in this network because we only took top 10% ranked genes in the global PPI network. As shown in Figure 2, six of the seven targets are located in the inner core of the SpA PPI network, and only one situates at the outer layer, suggesting that the drugs may interfere with the disease by acting on proteins in the core.

To explore the implications of this PPI network to SpA, we conducted gene ontology (GO) analysis. We used the p-value to quantitatively measure whether this PPI network is statistically significantly enriched with genes of a specific Gene ontology (GO) term. In Table 1 we listed the most significantly enriched GO terms presented in the network, with p-values smaller than 0.001. It can be seen that proteins in this network are significantly involved in biological processes of immune system, such as the regulation of granulocyte macrophage, natural killer cell proliferation, as well as the activation of leukocytes, lymphocytes and T cells, in consistent with the immune-mediated feature of SpA.

**Table 1:** Selection of the most significantly enriched GO terms in the SpA PPI network

GO ID	GO term	Total genes	Mapped genes
0032725	positive regulation of granulocyte macrophage colony-stimulating factor production	5	5
0032819	positive regulation of natural killer cell proliferation	7	6
0002376	immune system process	2239	144
0006950	response to stress	3586	151
0051716	cellular response to stimulus	6383	179
0010941	regulation of cell death	1549	91
0042127	regulation of cell proliferation	1402	81
0002694	regulation of leukocyte activation	404	58
0051249	regulation of lymphocyte activation	351	56
0050863	regulation of T cell activation	287	49

**Figure 3:**
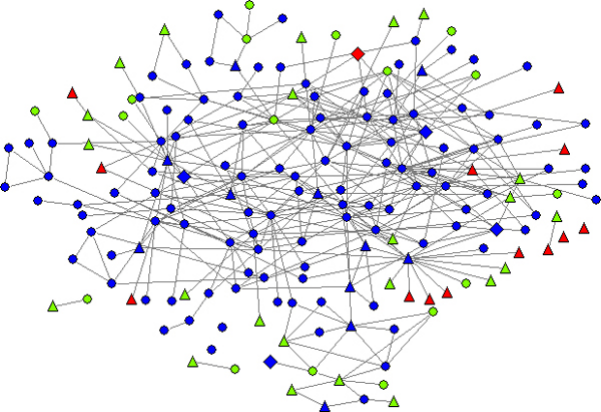
**A subnetwork of the SpA PPI network involved in innate immune response**. Blue, green and red nodes represent the inner core, the medium and outer core layer of the SpA PPI network in Fig.2, respectively. Triangles are seed genes in Figure 2.

The immune system process protects human beings against diseases with increasing specificity. The innate immune system provides an immediate, but non-specific response to invading pathogens; and the adaptive immune system, which is activated by the innate response in case those pathogens successfully evaded the response, adapts its response to improve its recognition of the pathogen. The innate immune response is important in the initiation of, and interplay with, the adaptive immune response. To investigate the association between the innate immune response and SpA, we constructed a subnetwork of the SpA PPI network involved in the innate immune response, by mapping proteins of the SpA network onto the PPI network of human innate immune response constructed from the InnateDB database [40] (see Figure 3). This subnetwork includes about half of the nodes of the SpA network, suggesting that the dysfunction of the innate immune system could be associated with the development of SpA. As can be seen in Figure 3, the innate immune subnetwork is significantly enriched with core nodes and non-seed nodes of the SpA PPI network (p-values are less than 0.001). This observation indicates that a very large fraction of proteins identified by our algorithm (non-seed nodes) are involved in the innate immune response, suggesting that in the human genome PPI network, the known SpA active proteins (seed nodes in our study) are close to a common group of innate immune proteins. Thus our approach reveals the important role of innate immune system in the initiation and development of SpA.

**Figure 4:**
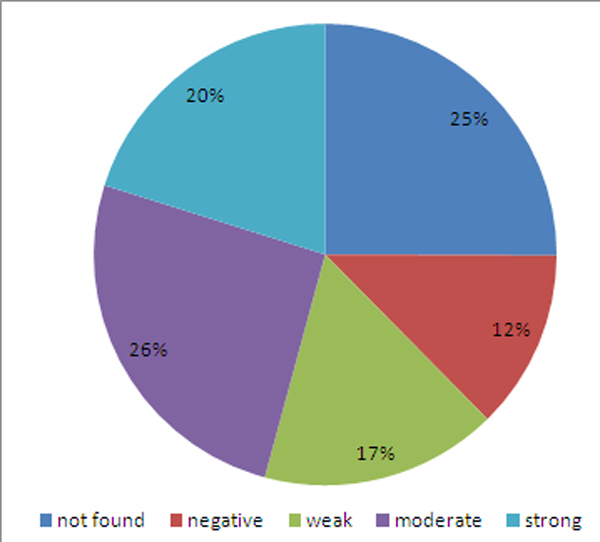
**Distribution of abundance levels of proteins of the SpA PPI network in hemopoietic cells of bone marrow**.

SpA injures the spine and the sacroiliac joints. It has been known that any kind of damage to the bone is mediated by osteoclasts, which are of hemopoietic cell origin [41]. To explore the tissue specificity of the SpA PPI network, considering that the interaction of two proteins cannot occur in a tissue if one of the proteins is not expressed in this tissue [42], we mapped proteins in the SpA PPI network onto the tissue specific protein expression dataset downloaded from the Human Protein Atlas portal [43]. A total of 275 out of the 367 proteins were identified in hemopoietic cells of bone marrow. As shown in Figure 4, the intensity and quantity of 63% proteins in this network could be detected in hemopoietic cells of bone marrow with abundance levels varying from weak to strong, suggesting a correlation of this network to bone pathogenesis.

### Pathways associated with SpA

To identify SpA-relevant biological processes, we mapped the 380 SpA associated genes onto the KEGG and Biocarta pathways, respectively. We used p-value to measure if a pathway is more likely affected by SpA associated genes (see Method part). Given significance level α = 0.05, we found that a total of 40 KEGG pathways are significantly enriched with genes in this group (see Additional file 1, Table S1). Similar pathways in the Biocarta database are also enriched with SpA associated genes. In Additional file 1, Table S2 we just listed four SpA gene enriched pathways included in the Biocarta database but not in the KEGG database.

A central feature of SpA is inflammation, one of the first responses of the immune system to infection or irritation. As listed in Additional file 1, Table S1, SpA is related to a large fraction of pathways in immune system. Some other pathways, although not classified into immune system in the KEGG database, have been known to be highly associated with the function of immune response, such as apoptosis [44], MAPK signaling pathway [45], and cell adhesion molecule interactions [46]. Specifically, Additional file 1, Table S1 includes several pathways related to pathogen recognition and inflammatory signalling in innate immune defences, in which the most important one is the Toll-like receptor (TLR) signalling pathway. The innate immune system relies on pattern recognition receptors (PRRs) to detect distinct pathogen-associated molecular patterns (PAMPs). Upon PAMP recognition, PRRs trigger a number of different signal transduction pathways. The pathways induced by PRRs ultimately result in the expression of a variety of proinflammatory molecules, such as cytokines, chemokines, cell-adhesion molecules, and immunoreceptors, which together orchestrate the early host response to infection, mediate the inflammatory response, and also bridge the adaptive immune response [46] together. The family of TLRs is the major class of PRRs [46]. The association of TLR2 and TLR4 with SpA has been reported [47]. It was noticed that three major signaling pathways were responsible for mediating TLR-induced responses including NF-kB, mitogen-activated protein kinases (MAPKs), and IFN regulatory factors (IRFs) [46], while we found that the two pathways, MAPKs and NF-kB, which play central roles in induction of a proinflammatory response, are involved in SpA. The tumor necrosis factor (TNFa) is an important upstream protein of the NF-kB pathway, which binds to its receptor to recruit TNF receptor death domain (TRADD) and thus activates NF-kB. TNF inhibitors have been proven highly effective for the treatment ofSpA [1]. In addition, we also found that SpA is associated with some proinflammatory molecule involved pathways, such as the chemokine signaling pathway, natural killer-cell mediated cytotoxicity, Fc epsilon RI signaling pathway, and cell-adhesion molecules interaction. These pathways indicate the process of innate immune response in the progress of SpA. On the other hand, it is known that B and T lymphocytes are responsible for the adaptive immune response [48]. Supplementary Table S1 shows the association of B and T cell receptor signalling pathways with SpA, implying their function in the adaptive immune response in SpA. In fact, it has been known that both the innate and adaptive immune responses are involved and interdependent with each other in SpA [49].

Another prominent feature of SpA is new bone formation; meanwhile bone loss is also a common finding in SpA [50]. As can be seen in Additional file 1, Table S1 and S2, SpA is associated with osteoclast differentiationand bone remodelling pathways, biological processes that maintain bone density and structure through a balance of bone resorption by osteoclasts and bone deposition by osteoblasts. Both ossification and osteoporosis symptoms of SpA are consequences of an imbalance in the regulation of these two sub-processes of bone remodelling. It is known that the WNT pathway regulates the balance between osteoclast and osteoblast function [51], verifying our result that SpA is associated with the WNT pathway (see Additional file 1, Table S1). In Additional file 1, Figure S1 we show SpA associated genes involved in the osteoclast differentiation pathway. Only one SpA causative gene from the OMIM database and three SpA active genes from the proteomics and microarray experiments appeared in this pathway, whereas a great fraction of genes involved in this network were predicted by our algorithm.

**Figure 5:**
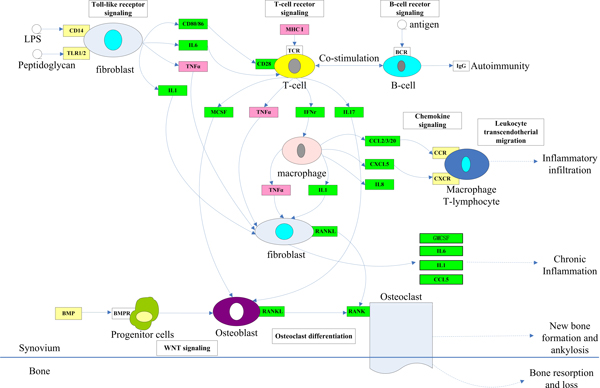
**Pathway of SpA**. Pink: Omim genes; Yellow: genes differentially expressed in the gene expression or proteomics experiments; Green: genes predicted by our algorithm.

Finally, integrating the two features of SpA with genes and pathways identified by our algorithm, we sketched an overview of biological processes involved in the development of SpA (Figure 5). Some SpA pathogenic factors, which remain unclear yet [52], on the one hand, trigger pathways of innate and adaptive immune responses to produce proinflammatory molecules, leading to inflammatory response. On the other hand, they also trigger progenitor cells, leading to unbalanced bone remodelling. Different pathways regulate these two aspects of pathological processes of SpA and interact with each other, in which some cytokines such as TNF and ILs, also known as SpA causative genes, play pivotal roles.

## Conclusions

We have extracted data related to SpA - known SpA causative genes from the OMIM database, proteomic experiments from literature, and microarray experiments from the GEO database. Using these genes as seeds, we developed a Katz'-centrality based index, *s*-score, to rank genes in the human PPI network. Then we considered 380 top-ranked genes as associated with SpA with high possibility. Based on these genes, we constructed a PPI network and identified potential pathways associated with SpA. The PPI network exhibits a core-periphery topology, in which most seed genes are located at the periphery part, while the inner core aggregates the non-seed genes enriched with innate immune genes and drug targets for SpA, suggesting that the core could be the modulating center of the network. The pathways we have discovered in this way represent the common knowledge of SpA, i.e., that it is an immune-mediated inflammation. Our data also reflect that an imbalanced bone modeling caused new bone formation and bone loss. We also illustrated the interplay between inflammation and bone injure. This network approach represents an alternative method for analyzing the complex effects of candidate genes related to complex diseases.

## Materials and methods

### Collection of SpA-associated genes

We collected genes associated with SpA from three resources as follows:

(1) The Online Mendelian Inheritance in Man (OMIM) database [30]: The OMIM database contains information on all known diseases and associated genes. We searched the database with a keyword "Spondyloarthropathy" and found 8 causal genes: HLA-B, TNFA, IL23R, CYP2D6, TNFSF13, TNFSF13B, B2M and COL2A1, in which gene CYP2D6 does not have an Entrez gene ID. The other seven genes are used as SpA-causative genes in this study.

(2) Proteomic experiment results: quantitative proteomics approaches were applied to investigate changes in protein expression in AS (the most common prototype of SpA) monocytes in comparison with healthy controls [53]. We used the 42 genes (Entrez ID) whose encoded proteins were differentially expressed [53] as potentially active genes in SpA.

(3) The NCBI Gene Expression Omnibus (GEO) database: we searched the GEO database and found 2 microarray experiments related to SpA: GSE1402 (Affymetrix U95Av2) and GSE18781 (AffymetrixHG_U133_Plus2). The GSE1402 experiment included a comparison of peripheral blood mononuclear cells (PBMC) from juvenile SpA with that of normal individuals, and the GSE18781 experiment was an investigation of peripheral blood cells from 18 subjects with SpA and 25 normal individuals. Samples in the GSE18781 experiment were processed as two separate sets at different times: 11 SpA + 12 control subjects in Set 1 and 7 SpA + 13 control subjects in Set 2. We treated the results of these two experiments as three separate datasets.

To integrate gene expression data from the two different platforms, we mapped the probe sets of the platforms to Entrez gene ID. This process yielded a set of 9448 genes common to the two platforms. For each gene in a dataset, we calculated the average expression level for probe sets associated with this gene, and filtered out genes whose mean expression ratios (SpA over control) in the two samples are greater than 0.67 (1/1.5) and less than 1.5. In the next step, we converted the expression value to its rank in the common genes [54]. Thereafter, a nonparametric two sample test, the Wilcoxon rank-sum test, was used to test if a gene is differentially expressed in the SpA and control samples and the p-value of the Wilcoxon rank-sum test was obtained. For such a large number of genes being simultaneously tested, the FDR [55] corrected p-values were used for screening differentially expressed genes. Given FDR level of 0.10, we found genes that are differentially expresses in the two samples for each of the three datasets. We then combined differentially expressed genes in the three datasets and identified 119 distinct genes potentially active in SpA.

Finally, the combination of disease causative genes from OMIM database, differentially expressed genes from the proteomics and microarray experiments yielded a total of 168 potentially SpA-active genes, which were used in a subsequent analysis.

It is noted that the SpA associated genes collected from the three resources have no overlap, which is likely due to the different levels of data origins. OMIM genes were collected from literatures focused on single gene or protein studies, while microarray and proteomic data were generated from genome-scale experiments at different levels. It is known that the activity of gene and protein is highly dynamic and can change rapidly in response to changes in internal and external environments. However, most current genome-scale experiments could not capture the entire dynamics but only take snapshots at single time points in specific experimental settings. This is why in most cases these data have little overlap. In order to get a better view of the disease pathology, we need to integrate different data resources.

### Candidate disease genes

We downloaded human gene location data from the ftp of NCBI MapViewer [56], which include the chromosomal locations and chromosomal base pair ranges of human genes. For each of the 7 known SpA-causative genes, we determined a set of *N *candidate genes, including this disease gene, which locate at, or near the cytogentic loci of the disease gene. Here, the number of candidate genes is about 100. If the chromosomal region where the disease gene located has more than 100 genes, we took 100 genes near the disease gene as candidates; otherwise, we took all genes in this region.

### Protein-protein interaction data

Weighted protein-protein interactions (PPI) of human beings were downloaded from version 8.3 of STRING [34]. STRING includes both physical and functional interactions integrated from numerous sources, including experimental repositories, computational prediction methods and public text collections; uses a scoring system to weigh the evidence of each interaction; and includes the interactions between 14532 proteins (Entrez gene ID) of human genome. We normalized the interaction scores in STRING to the area [0, 1] and represented the weighted PPI network as a matrix W.

Innate immunity-relevant human proteins and their interactions were downloaded from the InnateDB database [40] on April 25, 2011. Till the day we downloaded the data, this database includes 2310 human genes and 4819 interactions manually collected by literature review.

### Tissue specific protein expression data

Protein expression data in human normal tissue were downloaded from the web of the Human Protein Atlas [43]. Human Protein Atlas portal is a publicly available database including the spatial distribution and the relative abundance of proteins in 46 different normal human tissues and 20 different cancer types, as well as 47 different human cell lines. The protein abundance scales were combined into four levels: negative, weak, moderate and strong. We downloaded the file named normal_tissue.csv on September 16, 2011 and then extracted proteins expressed in hematopoietic cells of bone marrow and their abundance scales. This dataset includes 10798 gene-coded proteins (Entrez ID) in total.

### Pathway data

We downloaded pathway data from the FTP service of KEGG [57] (Kyoto Encyclopedia of Genesand Genomes) on June 21, 2011. The KEGG PATHWAY section is a collection of manually drawn pathway maps representing the information on the molecular interaction and reaction networks. The hsa_pathway.list file in this section includes a list of known proteins encoded by *H. sapiens*'s genome and the corresponding pathways in which these proteins are involved.

### k-core and k-core layer

**Figure 6:**
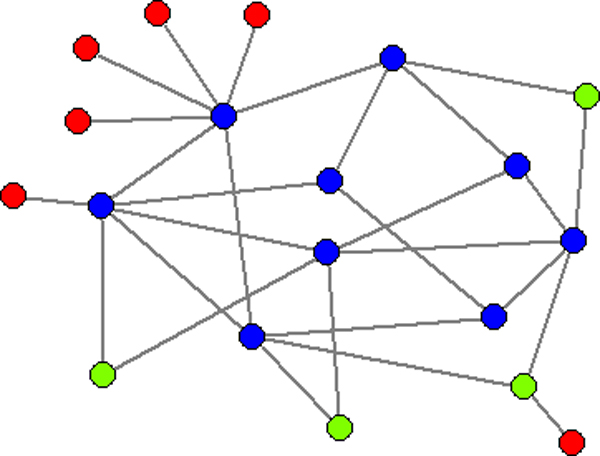
**Illustration of *k*-core and *k*-core layer**. The 1-core layer are the red nodes, the 2-core layer are green. The 1-core is the whole graph, the 2-core is subgraph consisting of green and blue nodes and the 3-core is the subgraph consisting of blue nodes.

The *k-*core of a graph is the maximal subgraph such that all of its nodes has at least *k *links within the subgraph [35,58]. The *k*-core layer *l_k _*is defined as the set of nodes that belong to *k*-core but not to *k*+1-core, i.e., *k*-core is the union of *k*+1-core and *k*-core layer. A *k*-core subgraph of a graph can be generated by recursively deleting the vertices from the graph whose present degree is less than *k*. This process can be iterated to gradually zoom into the more connected parts of the network. The higher-level core corresponds to more densely connected part of the network. See Figure 6 for an explanation.

### P-value

If we randomly draw *n *samples from a finite set, the probability of getting *i *samples with the desired feature by chance obeys the hypergeometric distribution with the following probability mass function:

(4) $$f\left(i\right)=\frac{\left(\begin{array}{c} k\\ i \end{array}\right)\left(\begin{array}{c} N-K\\ n-i \end{array}\right)}{\left(\begin{array}{c} N\\ n \end{array}\right)}$$

Where *N *is the size of the set, *K *is the number of items with the desired feature in the set. Then the probability, defined as the P-value, of getting at least *k *samples with the desired feature by chance can be obtained as the following, using the hypergeometric cumulative distribution function (CDF),

(5) $$p=1-\sum_{i=0}^{k-1}\text{f}\left(\text{i}\right)=1-\sum_{i=1}^{k-1}\frac{\left(\begin{array}{c} K\\ i \end{array}\right)\left(\begin{array}{c} N-K\\ n-i \end{array}\right)}{\left(\begin{array}{c} N\\ n \end{array}\right)}$$

Given a significance level α, a p-value smaller than α demonstrates a low probability that the items with the desired feature are chosen by chance. Hence this p-value can be used to measure whether the *n *samples drawn from the set is more enriched with items of the desired feature than would be expected by chance [59].

## List of abbreviations

AS: ankylosing spondylitis; MAPKs: mitogen-activated protein kinases; PPI: protein-protein interaction; PRRs: pattern recognition receptors; SpA: Axial spondyloarthropathy; TLR: Toll-like receptor; TNFa: tumor necrosis factor; TRADD: TNF receptor death domain.

## Competing interests

The authors declare that they have no competing interests.

## Authors' contributions

JZ and PH conceived the study, designed the experiments and wrote the manuscript. JZ, JC and THY performed the experiments and analyzed the data. All authors read and approved the final manuscript.

## Supplementary Material

Additional file 1 **Additional file for "Insights into the pathogenesis of axial spondyloarthropathy from network and pathway analysis"**. Supplementary material for this paper. Click here for file
