# Supplementary material for: Insights into the pathogenesis of axial spondyloarthropathy from network and pathway analysis
Source: BMC Syst Biol. 2012 Jul 16;6(Suppl 1):S4. doi: 10.1186/1752-0509-6-S1-S4 (PMC3403611; doi:10.1186/1752-0509-6-S1-S4)
Supplement: Additional file 1 — Additional file for "Insights into the pathogenesis of axial spondyloarthropathy from network and pathway analysis". Supplementary material for this paper. [file 1752-0509-6-S1-S4-S1.pdf]

Additional file for "Insights into the pathogenesis of axial spondyloarthropathy from network and pathway analysis"

Contents

Table S1 KEGG pathways significantly enriched with SpA-associated genes.
Table S2 Selection of biocarta pathways significantly enriched with SpA-associated genes.
Figure S1 SpA associated genes involved in the osteoclast differentiation pathway.

Table S1 KEGG pathways significantly enriched with SpA-associated genes.

**Pathway class**	**Pathway name**	**Total genes**	**Mapped gene**
Cell Communication	Focal adhesion	200	46
	Adherens junction	73	19
	Tight junction	132	15
	Gap junction	90	13
Cell Growth and Death	Apoptosis	86	27
	p53 signaling pathway	69	10
Cell Motility	Regulation of actin cytoskeleton	214	37
Development	Osteoclast differentiation	128	52
	Axon guidance	130	14
	Dorso-ventral axis formation	25	6
Endocrine System	Insulin signaling pathway	138	25
	GnRH signaling pathway	101	20
	Progesterone-mediated oocyte maturation	87	18
	Adipocytokine signaling pathway	69	17
	Melanogenesis	101	13
	Aldosterone-regulated sodium reabsorption	42	12
Immune System	Chemokine signaling pathway	189	62
	Natural killer cell mediated cytotoxicity	141	45
	Toll-like receptor signaling pathway	102	45
	T cell receptor signaling pathway	108	43
	Leukocyte transendothelial migration	116	42
	Hematopoietic cell lineage	88	35
	Fc epsilon RI signaling pathway	79	31
	B cell receptor signaling pathway	75	26

	Fc gamma R-mediated phagocytosis	95	25
	NOD-like receptor signaling pathway	59	22
	RIG-I-like receptor signaling pathway	71	19
	Antigen processing and presentation	78	16
	Intestinal immune network for IgA production	49	16
Signal Transduction	Cytosolic DNA-sensing pathway	62	13
	Jak-STAT signaling pathway	155	55
	MAPK signaling pathway	272	44
	ErbB signaling pathway	87	29
	VEGF signaling pathway	76	23
	Wnt signaling pathway	152	19
	mTOR signaling pathway	52	13
	TGF-beta signaling pathway	85	12
	Phosphatidylinositol signaling system	78	10
Signaling Molecules and Interaction	Cytokine-cytokine receptor interaction	275	80
	Cell adhesion molecules (CAMs)	135	21

Table S2 Selection of biocarta pathways significantly enriched with SpA-associated genes.

**Pathway name**	**Total genes**	**Mapped genes**
Bone Remodelling	13	6
Cytokines and Inflammatory Response	26	17
NF-kB Signaling Pathway	22	13
TNFR2 Signaling Pathway	17	8

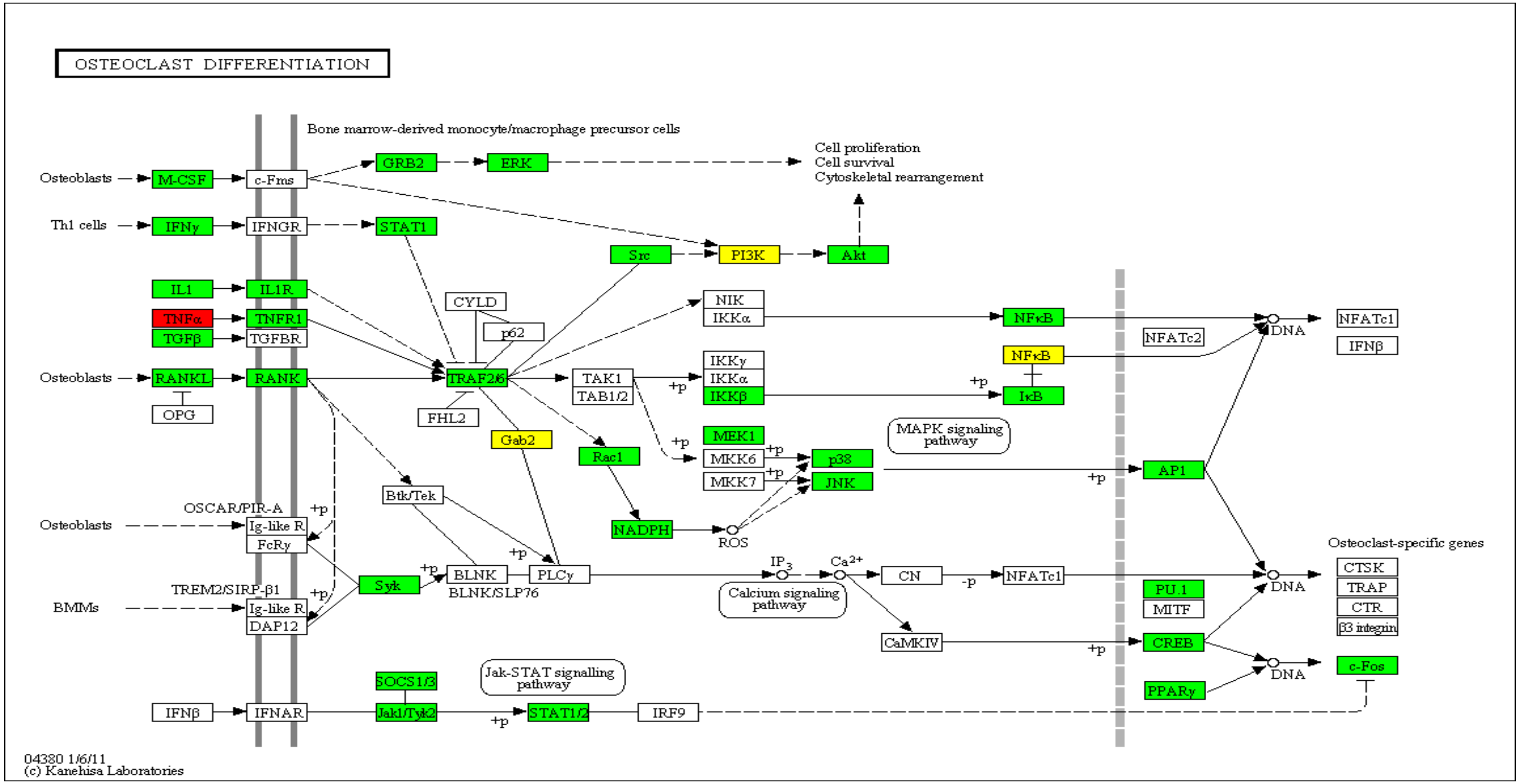

Figure S1 SpA associated genes involved in the osteoclast differentiation pathway. Red: Omim genes; Yellow: genes differentially expressed in the gene expression or proteomics experiments; Green: genes predicted by our algorithm. Original pathway map was downloaded from the KEGG website.
